# “The greedy I that gives”—The paradox of egocentrism and altruism: Terror management and system justification perspectives on the interrelationship between mortality salience and charitable donations amid the COVID‐19 pandemic

**DOI:** 10.1111/joca.12381

**Published:** 2021-05-31

**Authors:** S. Venus Jin, Ehri Ryu

**Affiliations:** ^1^ NU‐Q Communication Department Northwestern University in Qatar Education City Doha Qatar; ^2^ Department of Psychology Boston College Chestnut Hill Massachusetts USA

**Keywords:** charitable donations, consumer well‐being, COVID‐19 pandemic, mortality salience, system justification theory, terror management theory

## Abstract

Why do people give and help others in face of their own mortality salience? The existential struggle with the awareness of death impacts the gamut of human cognition, emotion, and behavior. This multi‐method research (∑*N* = 1,219) explains the psychosocial impact of COVID‐19‐related mortality salience on altruism. Drawing from terror management theory, two studies tested death‐thought accessibility, mortality salience, and anxiety buffer hypotheses. Study 1 (cross‐sectional survey), using structural equation modeling, confirms death anxiety and fear are predictors of powerlessness and materialism which, in turn, predict charitable donations. Study 2 (between‐subjects experiment) confirms the causal effects of COVID‐19‐induced mortality salience on altruism. Controlling income and socioeconomic status, people in the mortality salience treatment condition indicate greater monetary donations ($), ratio of prosocial (altruistic) to proself (egocentric) spending (%), donation of time (hour), monetary valuation of time (hourly rate = $/hour), and economic value of donated time (hourly rate*hour) than the controls. These effects are mediated by powerlessness. Moderating effects of relevant individual difference factors are significant: the greedier, more selfish, narcissistic, materialistic, and system‐justifying the donor is, the higher monetary donations, volunteer time, and perceived value of donated time are, only when the COVID‐19‐induced mortality is made salient but not in the controls. Environmental and dispositional factors jointly influence vulnerability to mortality salience. The paradox of egocentrism and altruism, as an evolutionarily adaptive protective buffer against existential insecurity for social and cultural animals, can help revitalize resilience, thus shedding some lights on the sociopsychological mechanism of consumers' subjective well‐being. Implications for consumer affairs, social marketers, and policymakers are discussed.

*We make a living by what we get. We make a life by what we give*.

Sir Winston S. Churchill.

## 1 INTRODUCTION

The rapid spread of the Severe Acute Respiratory Syndrome Coronavirus 2 (SARS‐CoV‐2) has resulted in the pandemic of the novel coronavirus disease 2019 (COVID‐19) (Segars et al., 2020). The crisis has affected everyday lives of billions around the globe through mortality, job losses, economic crises, shelter‐in‐place orders, border shutdowns, school closures, social distancing, and more. As of February 9, 2021 cumulative Coronavirus cases in the US. grew to more than 26.8 million and the death toll surpassed 462,037 (CDC). Among a wide array of valuable insights social and behavioral sciences can provide for managing the COVID‐19 pandemic and explaining its impacts (Van Bavel et al., 2020), the current study particularly focuses on consumers' charitable giving and prosocial (altruistic) versus proself (egocentric) spending as coping strategies amid the pandemic.

## 2 THE PARADOX OF SELFISHNESS AND ALTRUISM UNDER DEATH THREAT

The psychosocial responses of the general population toward epidemics include fear, anxiety, depression, irritability, sense of isolation, stigmatization, and so on (Sim et al., 2020). One notable phenomenon observed during the earlier stage of the COVID‐19 pandemic was panic buying exemplified with toilet paper hoarding in many countries, which could be interpreted as a selfish way (i.e., proself spending) to cope with insecurity, uncertainty, and loss of control (Arafat et al., 2020) or as a social dilemma of the inherent conflict between consumers' egocentric self‐interest and altruistic motivation for the good of the larger society as a whole (Boone et al., 2010; Cruickshank, 2020). Thus, death anxiety and consequent existential insecurity can bring out the selfishness and greed in people.

Paradoxically, times of turmoil simultaneously have brought out the best in people (Woods, 2020) as philanthropists, celebrities, ordinary individuals, firms, and institutions have donated their money and time generously (i.e., prosocial spending) in combating the global spread of the COVID‐19. For example, Michael Douglas, Gwyneth Paltrow, and Reese Witherspoon made monetary donations to the Frontline Responders fund and America's Food Fund, while Katy Perry has donated 10% from the sales of her fashion brands to Baby2Baby non‐profit organization to help provide essential items to children and families impacted by the COVID‐19 (Woods, 2020). Furthermore, a number of US medical students whose studies were interrupted by the Coronavirus outbreak have morphed their mission into helping the isolated elderly population highly vulnerable to the pandemic's dangers by doing grocery shopping, picking up prescriptions, delivering food boxes from food banks, or performing other errands for homebound seniors (Sewelll, 2020). Thousands of healthcare professionals nationwide have volunteered to support hospitals, frontline workers, and others battling the Coronavirus pandemic (Manzoni, 2020).

## 3 PURPOSE OF THE PRESENT STUDY

Why do people give, help others, and engage in prosocial behaviors while their own lives are also at risk amid the COVID‐19 crisis? Terror management theory (Greenberg et al., 1986; Pyszczynski et al., 1999) is a foundational framework that is well positioned to probe this question. The current study aims to elucidate the psychosocial mechanisms of charitable giving (time and money) and prosocial spending on others versus proself spending on self under the COVID‐19 death threat, drawing from terror management theory. To achieve this research objective, two studies (one cross‐sectional online survey [Study 1] and one between‐subjects online experiment [Study 2]) were conducted as an attempt to provide empirical findings from mixed methods research with methodological triangulation.

## 4 STUDY 1 (SURVEY) SYNOPSIS: “THE POWERLESS AND MATERIALISTIC I THAT GIVES”

Using structural equation modeling of survey data, Study 1 aims to test associations among death‐thought accessibility, death anxiety, powerlessness, materialism, and charitable giving amid the COVID‐19 pandemic.

### 4.1 Theoretical underpinnings and hypotheses developments

#### 4.1.1 Terror management theory

Humans, like all other forms of life, have a fundamental orientation toward self‐preservation and survival in the service of genetic replication (Solomon et al., 2003). Unlike other organisms, however, humans possess the intellectual capacity to understand the finite nature of life, knowing “the curtain will fall” (Arndt et al., 2005, p. 192). This prospect of dying is an impetus for managing and coping with existential anxiety (Hayes et al., 2008). People strive to resolve the conflict between their biologically and evolutionarily hardwired desire to survive and their rational awareness of inevitable death (Becker, 1975). According to terror management theory, this existential insecurity is managed by socially constructed beliefs that furnish people with “a sense of meaning, self‐worth, predictability, and a focus away from animal nature” (Hayes et al., 2008, pp. 611–612).

Terror management theory makes important assumptions about evolutionary adaptations (Greenberg et al., 1997). The evolutionary adaptations in response to terror and threat encompass (a) desire for self‐preservation, which is shared with other animals, resulting in “annihilation anxiety” and (b) intellectual and cognitive capacity, which is unique to humans, resulting in “awareness of the inevitability of death” (Greenberg et al., 1997, p. 71). The fear of death is rooted in an instinct for self‐preservation (Greenberg et al., 1986) and the “combination of an instinctive drive for self‐preservation with an awareness of the inevitability of death creates the potential for paralyzing terror” (Van den Bos et al., 2005, p. 92). In an attempt to minimize the paralyzing anxiety that arises from death cognitions, individuals adopt attitudes and behaviors that may bolster their self‐esteem and reinforce their worldview, thus giving meaning to their existence, symbolically extending their life into the future, and alleviating immediate feelings of anxiety (Frietze & Cohn, 2018; Greenberg et al., 1997). People develop an “anxiety buffering system” (Zaleskiewicz, Gasiorowska, Kesebir, Luszczynska, & Pyszczynski, 2013, p. 56) that helps protect them against existential fragility and furnishes them with psychological equanimity (Pyszczynski et al., 2010; Zaleskiewicz et al., 2015). To the extent that this anxiety buffer, which is a social psychological structure consisting of people's cultural worldviews and their self‐esteem, provides protection against death threats, inducing mortality salience increases their need for this buffer (Dechesne et al., 2003; Van den Bos et al., 2005).

#### 4.1.2 Death‐thought accessibility, mortality salience, and anxiety buffer hypotheses

Study 1 aims to not only test the three individual local‐level hypotheses proposed by terror management theory but also propose an integrative global‐level model composed of sequential paths indicating these hypotheses in the contemporary context of the COVID‐19 pandemic and charity donations: (a) death‐thought accessibility; (b) mortality salience; and (c) anxiety buffer hypotheses (Hayes et al., 2008).

Death‐thought accessibility is an indicator of deficient terror management (Helm et al., 2019) and is usually measured via a word‐stem completion task or a lexical decision task (Hayes et al., 2010). Death‐related thoughts produce different effects on cognition and behavior when they are in current focal attention (i.e., conscious proximal defense) and when they are on the fringes of consciousness (i.e., unconscious distal defense) (Pyszczynski et al., 1999, 2000). People cope with unconscious death‐related thoughts with increased defense of their cultural worldview (Pyszczynski et al., 1999).

According to the coping strategy literature compiled by North American Nursing Diagnosis Association‐International (NANDA‐I), death anxiety is defined as “vague uneasy feeling of discomfort or dread generated by perceptions of a real or imagined threat to one's existence” (Herdman, 2015, p. 356). In the nursing and coping strategy literature, powerlessness is defined as “the lived experience of lack of control over a situation, including a perception that one's actions do not significantly affect an outcome” (Herdman, 2015, p. 370). Consumer vulnerability is commonly conceptualized as a state of *powerlessness*, which arises from an interaction of individual characteristics, individual states, and external conditions (Baker et al., 2005; Huang et al., 2020). Mortality salience, which is indicated by death‐thought accessibility, fear, and death anxiety, can be associated with powerlessness. Amid the COVID‐19 pandemic, people may feel powerless due to the “perception that one's own action will not significantly affect an outcome” and the “perceived lack of control over a current situation or an immediate happening” (Braga & Cruz, 2009, p. 1064) during the crisis. Thus, this feeling of powerlessness is associated with and predicted by mortality salience (H1a and H2a), since death anxiety and fear of death make people vulnerable to feelings of being devoid of strength or resources as well as lead people to realize their lack of control over the life‐threatening environment (Herdman, 2015).

Furthermore, unconscious trepidation about death triggers a coping mechanism that entails the activation of dominant cultural beliefs and values (Becker, 1973; Rindfleisch & Burroughs, 2004). Worldview defenses might result from unconscious thought of death (Hayes et al., 2010). Most notably, fear of death has been identified as a possible antecedent of materialism (Christopher et al., 2006). Materialism, as one value within capitalistic cultures (Johnson et al., 2005), refers to the pursuit of material possessions or wealth (Richins & Dawson, 1992). The literature has viewed materialism as the culturally approved means of attempting to boost an individual's self‐worth to escape from negative thoughts or to avoid forms of insecurity (Donnelly et al., 2016; Elphinstone & Whitehead, 2019). Kasser and Sheldon (2000) found that mortality salience increases materialism. The fear associated with mortality salience leads people to lean on materialism as a means of coping with the ultimate fear (H1b and H2b), as the acquisition and possession of material objects is a pervasive cultural value in capitalistic societies (Arndt, Solomon, Kasser, & Sheldon, 2004). Therefore, it can be hypothesized that death‐thought accessibility, fear, and death anxiety are predictors of powerlessness (H1a and H2a) and materialism (H1b and H2b) during the COVID‐19 pandemic.

*Death‐thought accessibility is a positive predictor of (a) powerlessness and (b) materialism during the COVID‐19 pandemic*.

*Fear and death anxiety are positive predictors of (a) powerlessness and (b) materialism during the COVID‐19 pandemic*.

**FIGURE 1:**
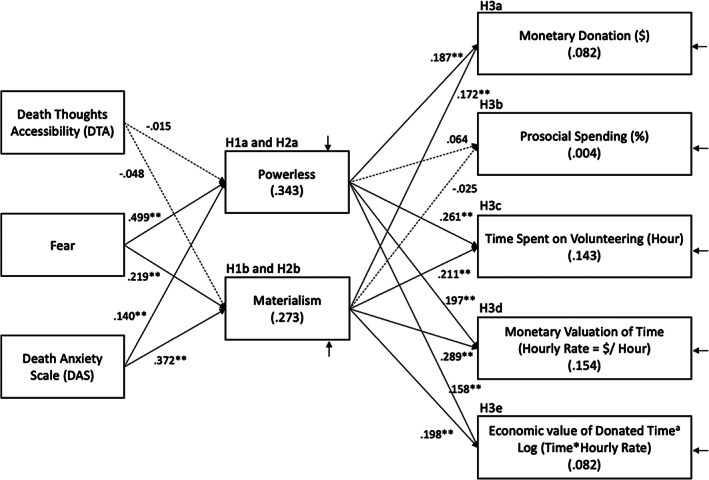
Study 1 (survey): Estimated structural equation model (H1, H2, and H3). *N*= 624. Completely standardized estimates are shown. * *p* < 0.05; ** *p* < 0.01; *p* > 0.10 for dashed structural paths. The three exogenous variables (DTA, fear, DAS) are allowed to covary with one another (not depicted in the figure). The residual covariance is allowed among the five outcome variables (not depicted in the figure). The *R* squared values are shown in parentheses for the endogenous variables. ^a^Economic value of donated time was computed by log (time spent on volunteering * hourly rate)

Acting prosocially is one source of value that exists within the cultural worldview people hold on as an attempt to deal with death anxiety from mortality salience (Zaleskiewicz et al., 2015). Regardless of cultures and social systems, prosocial behaviors are widely endorsed and provide the key ingredients for buffer against death anxiety, including a sense of value, meaning making, relatedness, and self‐transcendence (Greenberg et al., 1997). Mortality salience increases desire for prosociality, which in turn leads to more generous and less selfish allocation of financial resources (Zaleskiewicz et al., 2015). Prior research shows that individuals donate more when mortality is made salient to them (Jonas et al., 2013) (H3a, H3b, and H3c). Mortality salience is inherently associated with the extent to which people think about their time, since the amount of remaining time people have in their finite lives only decreases and the reminders of this finite nature of lifespan lead people to value their time more (H3d and H3e). Inducing such “limited time perspective” can facilitate reassessment and realignment of people's value systems (Cozzolino et al., 2009, p. 399). Considerations of limited time remaining may inspire a reordering of life priorities such that people perceive other‐centered and prosocial endeavors with altruistic motives as more important than self‐centered and proself endeavors with egocentric motives (Midlarsky & Kahana, 1994). Ultimately, Study 1 proposes that charitable giving and prosocial spending during the COVID‐19 pandemic are evolutionarily adaptive coping strategies to buffer against death anxiety, as powerlessness and vulnerable materialism induced by mortality salience increase people's need for this buffer (H3). The proposed conceptual models integrating death‐thought accessibility, mortality salience, and anxiety buffer hypotheses are presented in Figure 1.

*Powerlessness and materialism associated with mortality salience are positive predictors of (a) monetary donation intention ($); (b) the ratio of prosocial/altruistic spending intention to proself/egocentric spending intention (%); (c) donation of time (hour); (d) monetary valuation of one*'*s time (hourly rate = $/hour); and (e) economic value of one*'s donated time (hourly rate * hour).

### 4.2 Method

#### 4.2.1 Participants and data collection

A total of 624 people (*N* = 624; *M*_Age_ = 41.294 [minimum 18; maximum 66]; *SD*_Age_ = 13.023; 53.686% females; 76.122% White Caucasians, 8.493% African Americans, 6.410% Asians, 4.968% Hispanic and Latino Americans; .961% Native Americans, Alaska Natives, and Hawaiians, and 3.044% Others) participated in Study 1 (cross‐sectional survey data collection) on Amazon Mechanical Turk. All the research materials including protocol, recruitment message, anonymous survey questionnaire, and informed consent form were reviewed by the research ethics committee (i.e., Institutional Review Board) and received IRB approval.

#### 4.2.2 Measures

##### Death‐thought accessibility

Death‐thought accessibility was measured via a word‐stem completion task by asking participants to fill in the missing letters to various word stems, some of which can be completed with neutral words (e.g., KI_ _ ED with KISSED, COFF _ _ with COFFEE, GRA_ _ with GRAPE, etc.) or death words (e.g., KI_ _ED with KILLED, COFF_ _ with COFFIN, GRA_ _ with GRAVE, etc.) (Greenberg et al., 1994) (intercoder reliability between two independent coders = 0.94; range = 0 [lowest] to 7 [highest]).

##### Fear, death anxiety, powerlessness, and materialism

Fear was measured with 7‐point semantic differential scales (α = 0.968; e.g., “afraid,” “terrified,” “threatened.” etc.). Death anxiety was measured with three items from the death anxiety scale (DAS) extended (Templer et al., 2006) (α = 0.872; e.g., “I am afraid of dying from a life‐threatening disease”), powerless was measured with three items (α = 0.925; e.g., “I cannot make an impact on my surroundings, even when I want to”), and materialism was measured with three items from the materialism scale (Belk, 1985) (α = 0.918; e.g., “I am bothered when I see people who buy anything they want”), using 7‐point Likert scales ranging from “strongly disagree” [1] to “strongly agree” [7].

##### Donations of money and time

Multiple dimensions of charitable giving were measured by asking participants to indicate (a) the actual amount of monetary donation ($) they are willing to make; (b) the ratio (%) of spending on others (prosocial/altruistic spending) to spending on self (proself/egocentric spending); (c) the amount of time (hour) they are willing to donate to a charity by volunteering; and (d) monetary valuation of their time (hourly rate = $/hour). Economic value of participants' donated time (hourly rate * hour) was calculated by the researchers by multiplying the amount of donated time (hour) by the perceived value (hourly rate = $/hour) of participants' own time participants indicated.

### 4.3 Results and brief discussion

The correlation matrix is presented in Table 1. Results from structural equation modeling analyses show that the hypothesized model fit the data well: Chi‐square = 62.990, *df* = 16; CFI = 0.980; RMSEA = 0.069, and; SRMR = 0.056. The estimated path coefficients were consistent with the hypotheses, except for the influence of death‐thought accessibility on powerlessness and materialism, and for the influence of powerlessness and materialism on the ratio of prosocial spending to proself spending, as presented in Figure 1. This cross‐sectional survey data confirm the association among death anxiety, mortality salience, powerlessness, materialism, charitable donations (time and money) during the COVID‐19 pandemic.

**TABLE 1:** Study 1 (survey): Means, *SD*, and correlations

	Mean	*SD*	Correlation										
			1.	2.	3.	4.	5.	6.	7.	8.	9.	10.	11.
1. DTA (death‐thought accessibility)	2.433	1.405											
2. Fear	2.840	1.727	0.177^***^										
3. DAS (death anxiety scale)	3.435	1.810	0.102^*^	0.559^***^									
4. Powerless	3.232	1.623	0.088^*^	0.575^***^	0.417^***^								
5. Materialism	3.407	1.820	0.029	0.419^***^	0.490^***^	0.350^***^							
6. Monetary donation ($)	29.425	29.158	0.001	0.338^***^	0.301^***^	0.247^***^	0.237^***^						
7. Prosocial spending (%)	32.518	25.074	0.019	0.138^***^	0.112^**^	0.055	−0.003	0.637^***^					
8. Volunteering time (hour)	2.580	2.791	0.030	0.393^***^	0.353^***^	0.334^***^	0.301^***^	0.589^***^	0.352^***^				
9. Perceived value of time: Monetary valuation of time (hourly rate = $/hour)	28.261	23.393	−0.002	0.357^***^	0.300^***^	0.297^***^	0.357^***^	0.514^***^	0.217^***^	0.488^***^			
10. Economic value of donated time (volunteering time * hourly rate)	2.740	2.837	0.001	0.305^***^	0.268^***^	0.227^***^	0.253^***^	0.533^***^	0.344^***^	0.729^***^	0.603^***^		
11. SES (socioeconomic status)	4.169	1.322	−0.004	0.152^***^	0.131^**^	0.087^*^	0.272^***^	0.314^***^	0.175^***^	0.352^***^	0.355^***^	0.359^***^	
12. Income	3.257	1.518	.016	−0.079^*^	−0.039	−0.123^**^	0.040	0.013	0.013	−0.034	0.045	0.051	0.367^***^

*Note:* N = 624. Eight observations have missing values on SES. Nine observations have missing values on Income. Economic value of donated time was calculated by log (Volunteering Time * Hourly rate). Perceived SES was measured with 7‐point scales: 1 (the lowest status) to 7 (the highest status). Income level was measured with 7‐point scales = 1 (less than $25,000); 2 ($25,000 ~ $49,999); 3 ($50,000 ~ $74,999); 4 ($75,000 ~ $99,999); 5 ($100,000 ~ $149,999); 6 ($150,000 ~ $199,999); 7 ($200,000 or more).

Abbreviations: DAS, death anxiety scale; DTA, death‐thoughts accessibility; Hourly rate, Perceived monetary value (monetary valuation) of one's own time.

^***^ *p* < 0.001; ^**^ *p* < 0.01; ^*^ *p* < 0.05.

## 5 STUDY 2 (EXPERIMENT) SYNOPSIS: “THE SELFISH, NARCISSISTIC, GREEDY, AND MATERIALISTIC I THAT GIVES UNDER DEATH THREAT”

Building upon the associations among the key variables relevant to terror management found in Study 1, Study 2 examined the causal effects of the COVID‐19‐induced existential insecurity on charitable donations. Study 2 further tests the three general hypotheses proposed by terror management theory: (a) death‐thought accessibility; (b) mortality salience; and (c) anxiety buffer hypotheses (Hayes et al., 2008), using a between‐subjects experimental design. Original mediation and moderation hypotheses are additionally proposed to advance terror management theory. More specifically, Study 2 examined (a) the effects of reminding people of their vulnerability to the COVID‐19‐induced threat on death‐thought accessibility and affective response (fear, mood, arousal, and powerlessness); (b) the effects of mortality salience on charitable giving (time and money), prosocial spending, materialistic pursuits, and system justification as well as the mediating effects of powerlessness; and (c) the moderating effects of dispositional personality and individual difference factors (selfishness, narcissism, greed, materialism, status consumption, and system‐justifying tendency), in accounting for the effects of environmental factors (the COVID‐19‐induced mortality salience) on charitable donations and prosocial spending as anxiety buffering coping strategies.

### 5.1 Theoretical underpinnings and hypotheses developments

#### 5.1.1 Death‐thought accessibility, fear, and death anxiety

Driven by the terror management theory (TMT), Nepomuceno and Laroche (2016) tested the effects of death thoughts on anti‐consumption lifestyles. After death thoughts, the adoption of an anti‐consumption lifestyle buffers the effects of death thoughts and consequent mortality salience on people's consumption‐related behavior (Nepomuceno & Laroche, 2016).

From an evolutionary perspective, fear of death provokes anxiety since it is incompatible with the organismic tendency to preserve life (Greenberg et al., 1986; Hayes & Schimel, 2018). Conceptually, death‐thought accessibility occurs when an individual's anxiety buffer, which should “function to keep death‐related cognitions and associated potential anxiety at bay,” is threatened (Helm et al., 2019, p. 2). Threats to the cultural view or self‐esteem bring death‐thoughts closer to consciousness, thus increasing death‐thought accessibility (Hayes et al., 2008). The COVID‐19‐induced mortality has put people's cultural worldview and self‐esteem in peril: the pandemic has been tremendously impacting the whole social, economic, and cultural system by posing a threat to virtually every sector of our society and the entire gamut of human relationship including health, medicare, education, economy, transportation, media, and communication, to name a few. Prior research shows low death‐thought accessibility immediately following mortality salience manipulation as an active suppression of such thoughts as opposed to a delayed increase in death‐thought accessibility from relaxation of the suppression that covaries with worldview defense following mortality salience (Solomon et al., 2004). According to the death‐thought accessibility hypothesis, mortality salience instigates immediate death‐thought suppression (Hayes et al., 2010). Conscious thoughts of death are defended against with proximal defenses, which entail active suppression of such thoughts (Pyszczynski et al., 1999). In line with Hayes et al. (2010), it can be predicted that when measured immediately after mortality salience induction, death‐thought accessibility would remain relatively low, presumably because participants are still engaged in active suppression of the death‐related content. Mortality cues in the mortality salience treatment condition, therefore, would decrease the accessibility of death‐thoughts, operationalized as the number of death‐related words generated in a word‐stem task (H1c). Thus, Study 2 tests if experimentally furthered mortality salience regarding the current crisis (via exposure to media content about the COVID‐19 pandemic) actually has main effects on increase in death anxiety (H1b), death‐thought accessibility (H1c), and affective responses including fear (H1a), powerlessness, mood and arousal (H1d).

*People in the COVID‐19‐induced mortality salience treatment condition will (a) feel higher fear; (b) feel higher death anxiety; (c) score lower on* death‐thought *accessibility; and (d) feel more powerless*, *sad*, *and aroused*, *compared to those in the no mortality salience control condition*.

#### 5.1.2 Mortality salience, time, money, and spending on self versus others

One method through which the impact of fear‐based emotional appeals on consumers' attitudes and behaviors is strengthened is by reminding consumers of their own mortality (Kareklas & Muehling, 2014). According to the mortality salience hypothesis, if a psychological structure functions to buffer awareness of death, inducing people to think of their death increases their need for this psychological structure since reminding people of their mortality increases their need for self‐esteem and their faith in the cultural worldview (Hayes et al., 2010). Making mortality salient causes people to become more generous and less selfish in their behavioral choice (Zaleskiewicz et al., 2015). When an individual's death and mortality are made salient, the amount of donations to a charity is correlated with the extent to which the individual's engagement in the donation domain is an important source of self‐esteem (Ferraro et al., 2005). Reminders of mortality generally encourage people to be more charitable to others because generous behavior helps people bolster the belief that they are valuable contributors to their culture (Jonas et al., 2013). Therefore, it can be hypothesized that experimentally manipulated COVID‐19‐induced mortality salience would increase people's monetary donations to a charity (H2a), the ratio of prosocial spending to proself spending (H2b), and the amount of time they are willing to donate through volunteering for a charity (H2c).

Time and money are both valuable resources but the two have different characteristics, in the sense that the amount of money one has may increase whereas the amount of time one has only decreases and is bounded by 24 hours in a day as well as by one's lifespan (Monga & Zor, 2019). Time and money are also different in terms of their perceived value and appropriateness as resources for donation. Asking people to donate time activates a distinct mindset compared to asking people to donate money, such that donations of time activate a mindset and goals related to emotional well‐being, whereas monetary donations elicit a mindset and goals associated with economic utility and value maximization (Liu & Aaker, 2008). People tend to perceive time donations to be more effortful and attribute more altruistic motive compared to monetary donations, thus implicating the power of time donations in increasing other‐serving, prosocial motive attributions (Langan & Kumar, 2019). Therefore, it can be expected that the COVID‐19‐induced mortality salience will not only increase people's perceived value of their own time (H2d and H2e) but also lead them to value their time more than their money (H2f). Altogether, these theoretical foundations and prior empirical findings guided the formation of the second hypothesis (H2).

*People in the COVID‐19‐induced mortality salience treatment condition will indicate greater (a) monetary donations ($) intention; (b) ratio of prosocial/altruistic spending intention to proself/egocentric spending intention (%); (c) donation of time (hour); (d) monetary valuation of their time (hourly rate = $/hour); (e) economic value of their donated time (hourly rate x hour); and (f) value their time more than their money*, *compared to those in the no mortality salience control condition*.

#### 5.1.3 Mediating effects of powerlessness

Mortality salience, as a trigger of existential insecurity, induces feelings of powerlessness. Feeling less powerful can lead people to indicate a higher willingness to spend money in order to restore feelings of power (Rucker & Galinsky, 2008, 2009). According to the powerlessness‐induced compensatory model of consumption, “the acquisition of status can increase one's felt sense of power.” (Rucker et al., 2012, p. 360). Donation behaviors can be seen as a coping strategy to restore individuals' lost sense of power (Mandel et al., 2017), in response to the heightened mortality salience during the COVID‐19 pandemic. High‐power leads people to place greater weight and value on the self, thereby increasing their willingness to spend on themselves (proself spending) over others, whereas low‐power increases people's dependence on others, thereby increasing their willingness to spend on others (prosocial spending) (Rucker et al., 2012). High‐power states make the self more focal, whereas low‐power states shift attention and value to others, thus inducing more altruistic monetary spending on others (Rucker et al., 2011), which suggests the mediating effect of powerlessness on the relationship between mortality salience and charitable donations (H3).

*The influence of the COVID‐19‐induced mortality salience* versus *no mortality salience on charitable donation intentions (time and money)*, *the ratio of prosocial/altruistic spending to proself/egocentric spending*, *and monetary valuation of time will be mediated by powerlessness*.

#### 5.1.4 Altruism, materialistic value, and system justification as anxiety buffers against the COVID‐19

Regardless of the level of mortality salience, spending money on others is associated with happiness (Dunn et al., 2008) and using money to benefit others pays off (Aknin et al., 2020; Dunn et al., 2014). Study 2 particularly tested the causal effects of experimentally activated COVID‐19‐induced mortality salience on the amount of charitable donations (money and time) and the ratio of prosocial to preself spending (H2). However, since cultural norms often encourage both generosity and the accumulation of material wealth simultaneously, the terror management literature on people's monetary behaviors and attitudes reveals seemingly contradictory results suggesting that mortality salience paradoxically leads to both generosity (H2, H4a, H4b, and H4c) and greed (H4d and H4e) (Jonas et al., 2002; Jonas et al., 2013; Kasser & Sheldon, 2000).

Generosity toward other people or prosocial causes can fulfill psychological needs such as a sense of power, which in turn could soothe concerns about inevitable death and existential fragility (Zaleskiewicz et al., 2015). One of the most prominent psychosocial responses people show when they need to protect themselves is seeking out others (Chan, 2017). From an evolutionary perspective, human altruism, reciprocity, and cooperation can be viewed as evolutionarily adaptive strategies (Schindler et al., 2013) since altruism can be driven by the need for self‐protecting individuals from dangerous situations, in order to survive eventually (Chan, 2017). Thus, to the extent pursuits of generosity and altruism as intrinsic values can augment materialistic striving and provide an avenue through which people can feel good about themselves, these intrinsic value systems manifested via prosocial behaviors (e.g., donating time and money, helping others, contributing to the community) may offer “protection from existential anxieties that terror management research indicates is necessary for psychological equanimity” (Arndt, Solomon, Kasser, Kennon, & Sheldon, 2004, p. 210). Autonomous motivations and the corresponding behaviors are primarily intrinsic in nature (Weinstein & Ryan, 2010) and, consequently, reflective of and congruent with one's values, desired self, goals, and aspirations (Leary et al., 2019; Ryan & Connell, 1989). Applying these assumptions to prosocial behaviors, intrinsically and autonomously motivated consumers are more likely to show positive well‐being behaviors especially when they believe that their actions have the potential to influence *others* (Leary et al., 2019). The extent to which a prosocial act is volitional predicts its effects on well‐being and psychological need satisfaction mediates this relation (Weinstein & Ryan, 2010). Integrating evolutionary perspectives on human altruism and motivational theories of prosocial behavior, Study 2 proposes that mortality salience increases psychological satisfaction derived from prosocial behavior such as prosocial spending (i.e., spending on *others*) (Zaleskiewicz et al., 2015) (H4a, H4b, and H4c).

Terror management theories of materialism propose that mortality salience also increases materialistic behavior and greed (Kasser & Sheldon, 2000). Making mortality salient increases materialism (Arndt, Solomon, Kasser, Kennon, & Sheldon, 2004). Being primed with death salience has been correlated with higher levels of materialistic value (Elphinstone & Whitehead, 2019; Rindfleisch et al., 2009). Since materialistic value is one of the central values in Western cultures, money earned and accumulated often symbolizes the extent to which a person is valued and capitalistic norms often encourage members of the society to try to earn more money than others (Jonas et al., 2013). Therefore, when mortality salience is induced, members of capitalist and materialistic cultures, where accumulation of wealth and consumption of goods are touted as paths to a successful and happy life, will increase their materialistic pursuits as a way of bolstering this worldview and reinforcing their belief that they are valuable members within this ideological framework (Kasser & Sheldon, 2000; Nepomuceno & Laroche, 2016). A large body of terror management research revealed the pursuit of materialistic value and consumption as potential death anxiety buffers in capitalist societies (Gasiorowska et al., 2018). Since “money serves the vital psychological function of soothing existential anxiety” (Zaleskiewicz, Gasiorowska, Kesebir, Luszczynska, & Pyszczynski, 2013, p. 55), the pursuit of materialistic value, as an existential anxiety buffer, may help people cope with negative feelings evoked by painful mortality salience (Gasiorowska et al., 2018) during the COVID‐19 pandemic (H4d). Likewise, feeling less powerful due to the reminders of mortality can also lead consumers to report a higher need for status and greater willingness to pay for high‐status products, in order to restore feelings of power (Rucker & Galinsky, 2008, 2009) and reinforce their sense of value in society (Mandel et al., 2017) (H4e).

According to system justification theory, “people exhibit system‐justifying tendencies to defend and rationalize existing social, economic, and political arrangements, sometimes at the expense of individual and collective self‐interest” (Jost, 2019, p. 263). Terror management theory converges with system justification theory on the point that maintenance of worldview is the fundamental component of anxiety buffering system and defense mechanisms (Landau et al., 2009): “anxiety buffering function” of worldviews proposed by terror management theory (Greenberg et al., 1997, p. 67) and the “palliative function of system justification” proposed by system justification theory (Jost, 2019, p. 273). System justification serves a palliative function in that it reduces anxiety, discomfort, and uncertainty (Jost & Hunyady, 2003). Mortality salience and death anxiety have been identified as antecedents of system justification (Jost & Hunyady, 2005). Existential awareness of inevitable death, anxiety arising from mortality concerns, and fear associated with the prospect of one's own death enhance system‐justifying beliefs among members of the society (Landau et al., 2004). Empirical research also supports the notion that a sense of powerlessness fosters system justification (Van Der Toorn et al., 2015). Powerlessness induced from the COVID‐19‐related mortality salience may foster system justification. It can be hypothesized, therefore, that the COVID‐19‐induced mortality salience may foster system justification (H4f).

*People in the COVID‐19‐induced mortality salience treatment condition will indicate higher (a) anticipated happiness felt through donation; (b) anticipated sense of power provided or restored through donation; (c) involvement with charitable giving; (d) materialistic value; (e) need for status; and (f) system justification*, *compared to those in the control condition*.

#### 5.1.5 Moderating effects of selfishness, narcissism, greed, materialism, and system justification

Interaction effects between individual difference factors and message contents using experimental conditions are the themes and methodological approach that should receive greater consideration when designing messages for PSA and campaigns (Smith & Stutts, 2006). To investigate the interaction effects of mortality salience and individual difference factors in consumer affairs deploying experimental design, Study 2 proposes the moderating roles of selfishness (H5a), narcissism (H5b), greed (H5c), materialism (H5d), status consumption tendency (H5e), and system‐justifying tendency (H5f), in accounting for the causal effects of the COVID‐19‐induced death salience on consumers' charitable donations and prosocial spending, guided by the theoretical foundations elaborated below.

People with a prosocial value orientation tend to maximize joint outcomes and are willing to cooperate, whereas people with a proself value orientation tend to pursue their self‐interest (Boone et al., 2010). Self‐interest can be harnessed to inform strategies to induce prosocial behavior such that simply alerting people to a health threat encourages them to engage in prosocial behaviors in order to reduce their own risk when self‐interest and collective benefit become aligned (Van de Vyver et al., 2018). Applying this assumption to the current COVID‐19 pandemic, helping prevent others from becoming infected or helping them recover through altruistic charitable giving and prosocial behaviors ultimately help protect the self, due to the highly contagious and viral nature of respiratory diseases. It can be expected that the correlation between selfishness and charitable donations will be negative in the control condition, such that selfishness decreases the amount of donations when there is no death threat. In contrast, the correlation between selfishness and charitable donations will be positive in the mortality salience treatment condition, such that selfishness increases the amount of donations because of a self‐protection motive prompting altruism (Chan, 2017). Therefore, it can be hypothesized that selfishness decreases charitable giving and prosocial spending when mortality is not made salient, whereas selfishness increases charitable giving and prosocial spending when the COVID‐19‐induced mortality salience is further activated experimentally (H5a).

From an evolutionary perspective, our intrinsic status as an organism makes us inevitably narcissistic as humans seek self‐preservation, longevity, and self‐importance in the face of mortality salience (Becker, 1973). Self‐aggrandizing and self‐centered narcissists can confirm their grandiose self by conspicuous donation behavior (Pilch & Gornik‐Durose, 2016) in order to create the desired image in the eye of others (Sedikides et al., 2007), thus boosting their self‐esteem through conforming to the cultural worldview and contributing to the social system as valuable members, especially when confronted with mortality salience. There is an underlying and paralyzing existential anxiety and this fear of death compels our yearning for the heroic (Kierkegaard, 1849/1989) (Kierkegaard, 1989). Thus, the pervasive pursuit of heroism as a means of denying the reality of death and *organismic narcissism* as a pursuit of self‐importance, defend us against the terror of death (Becker, 1973; Goodman et al., 2010). Therefore, it can be hypothesized that narcissism either decreases or does not affect donation intentions when mortality is not made salient, whereas narcissism increases donation intentions when the COVID‐19‐related mortality salience is made salient (H5b).

Kasser and Sheldon (2000) report that people behave with greater greed and financial ruthlessness in a simulation exercise during which they compete with others. From the terror management perspective, when cultural values of capitalistic competition are active, mortality salience catalyzes greedy behavior (Arndt, Solomon, Kasser, Kennon, & Sheldon, 2004) insofar as it provides a route to a sense of symbolic personal significance. However, just like the “threat of contagion arouses a cruel paradox of a crisis that calls for solidarity” in light of social distancing (Brody, 2020), the COVID‐19 pandemic provokes another cruel paradox of greed that calls for cooperation and altruism. The extremely contagious and viral nature of the pandemic may lead greedy people, who have the “desire to get more at all costs” (Mussel et al., 2018, p. 250), to become altruistic, as an attempt to prevent the spread of disease among other people to ultimately avoid infection and protect themselves, under explicit death threat. The COVID‐19‐induced death salience manipulation, therefore, may activate greedy people's altruism such that greedy people in the treatment condition show higher monetary donations, time donation, charity involvement, and prosocial spending than greedy people in the baseline control condition with no mortality salience manipulation. In contrast, less greedy people may be less sensitive to mortality salience, which suggests the moderating role of greed in determining the effects of the COVID‐19‐induced death salience on charitable giving and the ratio of prosocial spending to proself spending (H5c).

Materialism refers to the tendency to place accumulation of wealth and material possessions high within the hierarchy of values and treat it as an indicator of personal success and a source of happiness (Richins & Dawson, 1992). Mortality salience makes people more materially self‐interested only if this is in accordance with dispositionally salient norms (Jonas et al., 2013). Symbolic attitudes toward possessions and money moderate the effects of mortality salience on consumer behaviors, such that individuals allocating higher value to money are more reactive to mortality salience (Zaleskiewicz, Gasiorowska, & Kesebir, 2013), which suggests the moderating role of dispositional materialism within the society with certain norms in determining the effects of environmentally primed death salience. Low anti‐consumption individuals are more prone to consume when death is made salient (mortality salience condition), whereas frugals and voluntary simplifiers (high anti‐consumption individuals) have their propensity to consume unaffected by mortality salience (Nepomuceno & Laroche, 2016). The nature of mortality salience effects depends on the individuals' dispositionally and situationally relevant values (Arndt, Solomon, Kasser, & Sheldon, 2004). It can be reasonably hypothesized that highly materialistic people are more sensitive and vulnerable to mortality salience such that they indicate greater altruism, as an anxiety buffering coping strategy, when mortality is made salient than when mortality is not made salient. In contrast, less materialistic people are less vulnerable to death threats and may not show differential levels of donations and prosocial spending regardless of mortality salience (H5d).

Relevant research in the evolutionary framework describes conspicuous consumption as “an adaptive strategy, associated with a universal tendency for signaling features that might boost status” (Pilch & Gornik‐Durose, 2016, p. 103). Prior research also shows that materialism moderates the impact of mortality salience on impulsive tendencies toward luxury brands (Audrin et al., 2018), which suggests the role of status consumption tendency in determining the effects of mortality salience. Mortality salience leads consumers to excessively consume as a way to cope with thoughts of impending mortality (Mandel & Smeesters, 2008). Charitable giving, which could be perceived as a behavioral outcome of status or conspicuous consumption and an attribution of symbolic power of money to prosocial behavior, can function as an anxiety buffering coping strategy in face of mortality salience. Symbolic status obtained or superiority felt through luxury consumption helps restore a sense of power or control when confronted with existential insecurity (Mandel & Heine, 1999). Status consumption tendency may increase charitable donations and prosocial behaviors only when mortality is made salient and subsequently the need for an anxiety buffer is activated. Therefore, it can be expected that status consumption tendency may be boundary conditions for explaining the effects of reminders of mortality salience on charitable giving and prosocial spending (H5e).

Although terror management theory and system justification theory have distinct tenets about human motivation and often times assume different levels or units of analysis (individual, group, system‐level threats) (Anson et al., 2009; Cutright et al., 2011), both theories originated in an effort to explain social psychological defense mechanisms in response to a threat which heightens needs to defend and justify the worldview against the threat (Jost et al., 2004; Landau et al., 2009; Pyszczynski et al., 2008). Prior research empirically supports the interaction effects of death anxiety and existential motives on system justification (Hennes et al., 2012). The current research proposes the interaction effects of system‐justifying tendency and mortality salience on charitable giving, such that the more system‐justifying the donor is the greater charitable donations are, only when mortality salience is made salient, but not in the control condition (H5f). Altogether, these theoretical reasonings guided the formation of the moderation hypothesis (H5).

*The influence of the COVID‐19‐induced mortality salience* versus *no mortality salience on charitable donation intentions (money and time)*, *the ratio of prosocial/altruistic spending to proself/egocentric spending*, *monetary valuation of time*, *and involvement with charitable giving will be moderated by (a) selfishness; (b) narcissism; (c) greed; (d) materialism; (e) status consumption tendency; and (f) system‐justifying tendency*.

### 5.2 Method

#### 5.2.1 Design and participants

Study 2 employed a between‐subjects (mortality salience condition [exposure to the COVID‐19 video] versus control condition [no video]) experimental design. Experiments were implemented using the Amazon Mechanical Turk (MTurk) online panel in the United States: pretest (*N* = 108) and main test (*N* = 487; *M*_Age_ = 41.673 [minimum 18; maximum 66]; *SD*_Age_ = 12.892; 48.665% females; 74.949% White Caucasians, 11.294% African Americans, 5.544% Asians, 5.133% Hispanic and Latino Americans; 1.232% Native Americans, Alaska Natives, and Hawaiians, and 1.848% Others). Applying the MTurk qualification criterion (worker requirement/qualification: “Completed my survey already” “has not been granted”) to Study 2 main test (experiment), those MTurk panel members who already participated either in Study 1 (survey) or in the pretest for Study 2 (experiment) were automatically excluded during the recruitment process to ensure internal validity. All the research materials including protocol, recruitment message, anonymous survey questionnaire, manipulation stimuli, and informed consent form were reviewed by the research ethics committee and received IRB approval.

#### 5.2.2 Pretest and manipulation stimuli

A pre‐test (*N* = 108) was conducted to check manipulation success of the COVID‐19‐related mortality salience activation. In order to ensure that there is a significant difference between the treatment condition (the COVID‐19‐induced mortality salience) and the control condition (no mortality salience) with regard to the level of death anxiety, one pretest (experiment) was conducted among the Amazon MTurk workers in the United States. Participants were randomly assigned to one of the two conditions (*N*_Treatment Condition_ = 57; *N*_Control Condition_ = 51) and were measured on the level of death anxiety. The results of independent samples *t* tests confirmed that participants in the COVID‐19 mortality salience treatment condition showed higher death anxiety (*M*_Treatment Condition_ = 3.861, *SD*_Treatment Condition_ = 1.559) than those in the no mortality salience condition (*M*_Control Condition_ = 2.551, *SD*_Control Condition_ = 1.529), *t* = 4.399, *p* < 0.001. Based on this pretest result, which confirms successful manipulation checks, the same video was used in the main experiment to assess differential effects of the mortality salience treatment condition (the COVID‐19 video) versus the control condition (no video) on the proposed dependent variables.

#### 5.2.3 Measures

##### Fear, death anxiety, death‐thought accessibility, and powerlessness

Fear (α = 0.974), death anxiety (α = 0.874), death‐thought accessibility (intercoder reliability between two independent coders = 0.95; range = 0 [lowest] to 8 [highest]), and powerlessness (α = 0.914) were measured with the same items used in Study 1.

##### Donations of time and money

Multiple dimensions of charitable giving ($, hour, percentage, monetary valuation of time, and economic value of donated time) were measured using the same methodology and items.

##### Anticipated happiness and sense of power (consumer well‐being)

Anticipated happiness (α = 0.890; e.g., “To what extent do you think donating money to a charity will make you feel happy?”) and anticipated sense of power (α = 0.889; e.g., “To what extent do you think donating money to a charity will make you feel powerful?”) were measured with “anticipated happiness” and “sense of power provided” items from Rucker and Galinsky (2008).

##### Arousal and mood

Arousal (relaxed [1]–stimulated [7]) and mood (sad [1]–happy [7]) were measured with a single‐item 7‐point semantic differential scale.

##### Materialistic value, need for status, and system justification

Materialistic value was measured with three items from materialism value scale (α = 0.919; e.g., “The amount of material objects people own is a sign of their success”), need for status was measured with three items from Dubois et al.'s (2012) need for status scale (α = 0.913; e.g., “I have a desire to raise my relative position to others in the social hierarchy”), and system justification was measured with three items from Pratto et al.'s (1994) social dominance orientation scale (α = 0.902; e.g., “If certain groups stayed in their place, we would have fewer problems”).

##### Selfishness and narcissism

Selfishness was measured with nine items from the selfishness scale (Raine & Uh, 2019) (α = 0.878; e.g., “I care for myself much more than I care for others”). Narcissism was measured with narcissism scale (α = 0.931; e.g., “I like to be the center of attention”).

##### Greed, materialism, and status consumption

Greed (α = 0.914; e.g., “When I think about all the things I have, my first thought is about what I should have next”), materialism (α = 0.909; e.g., “I am bothered when I see people who buy anything they want”), and status consumption tendency (α = 0.948; e.g., “A product is more valuable to me if it has some snob appeal.”) were measured with three items from greed scale (Mussel et al., 2018), materialism scale (Belk, 1985), and status consumption scale (Eastman et al., 1999), respectively.

### 5.3 Results and brief discussion

#### 5.3.1 The effects of mortality salience on charitable donations (time and money)

**FIGURE 2:**
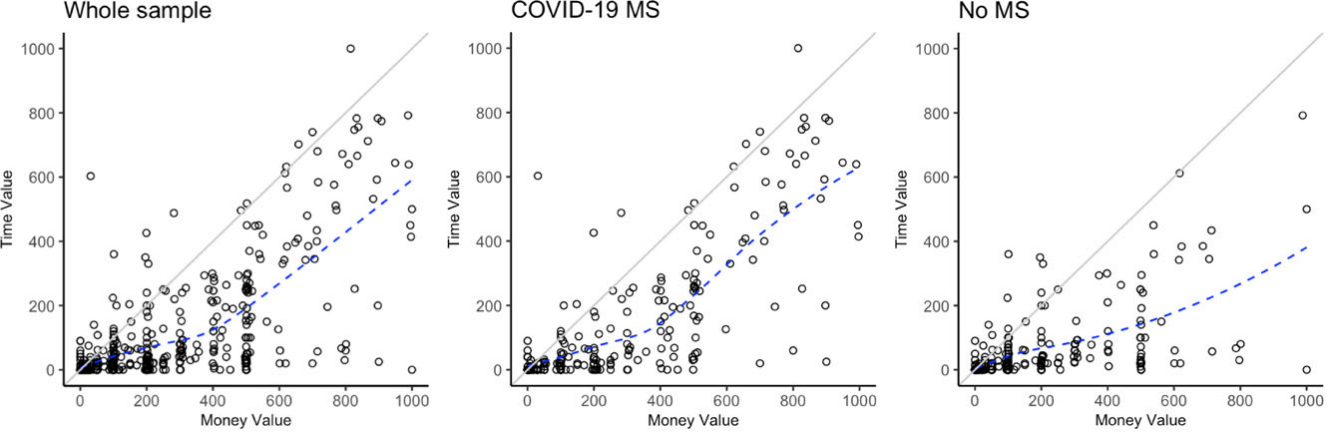
Study 2 (experiment): Histogram (H2e and H2f). X‐axis represents the amount of monetary donations (ranges from $0 to $1,000). Y‐axis represents the economic value of donated time (hourly rate * volunteering time)

Study 2 confirms that people donate more when there is a death threat such that participants in the treatment condition (exposure to media content about the COVID‐19‐related mortality salience) showed a higher amount of monetary donations (H2a), higher ratio of prosocial spending to proself spending (H2b), and a greater amount of donation of their own time (H2c), compared with those in the control condition (no mortality salience priming), as shown in the ANOVA Table (H1, H2, and H4: Table 2). Time (economic value of their donated time) versus money valuation comparison is presented in the histogram (H2e and H2f: Figure 2). Donors drive the most meaning from their donation decisions when they believe them to be personally costly and effortful (Olivola & Shafir, 2013).

**TABLE 2:** Study 2 (experiment): Means, *SD*, and ANOVA test of mean differences between conditions (H1, H2, and H4)

	Whole sample (*N* = 487)		Covid‐19 MS (*N* = 231)		No MS (*N* = 256)		Mean difference		
	Mean	*SD*	Mean	*SD*	Mean	*SD*	*F* (1, 485)	*p*	*d*
H1a: Fear	2.997	1.830	4.133	1.683	1.972	1.266	259.375	<0.001	1.462
H1b: DAS (death anxiety scale)	3.152	1.721	3.955	1.755	2.427	1.324	118.992	<0.001	0.990
H1c: DTA (death‐thought accessibility)	2.031	1.249	1.913	1.182	2.508	1.926	16.977	<0.001	0.372
H1d.1: Powerlessness	3.190	1.577	3.745	1.546	2.689	1.434	61.142	<0.001	0.710
H1d.2: Mood (sad)	3.567	1.020	3.446	1.105	3.676	0.925	6.236	0.013	−0.227
H1d.3: Arousal	2.667	1.122	3.004	1.109	2.363	1.046	43.047	<0.001	0.595
H2a: Monetary donation ($)	28.230	27.807	34.294	29.841	22.758	24.639	21.796	<0.001	0.424
H2b: Prosocial spending (ratio of spending on others to spending on self) (%)	31.967	24.760	34.368	24.764	29.801	24.604	4.158	0.042	0.185
H2c: Volunteering time (hour)	2.589	2.839	3.281	3.124	1.965	2.395	27.534	<0.001	0.476
H2d: Perceived value of time: Monetary valuation of time (hourly rate = $/hour)	27.402	21.894	31.450	25.103	23.750	17.804	15.468	<0.001	0.357
H4a: Happiness of giving	4.207	1.775	4.381	1.717	4.051	1.815	4.230	0.040	0.187
H4b: Sense of power provided or restored through donation	3.372	1.670	3.781	1.705	3.003	1.552	27.796	<0.001	0.478
H4c: Charity involvement	3.972	1.930	4.409	1.883	3.577	1.890	23.608	<0.001	0.441
H4d: Materialistic value pursuit	4.027	1.744	4.436	1.642	3.658	1.754	25.393	<0.001	0.457
H4e: Need for status	3.761	1.675	4.173	1.684	3.389	1.580	28.075	<0.001	0.481
H4f: System justification	3.221	1.803	3.696	1.888	2.793	1.610	32.390	<0.001	0.516

Abbreviation: *d*, effect size measure Cohen's d.

#### 5.3.2 Mediation effects of powerlessness

**FIGURE 3:**
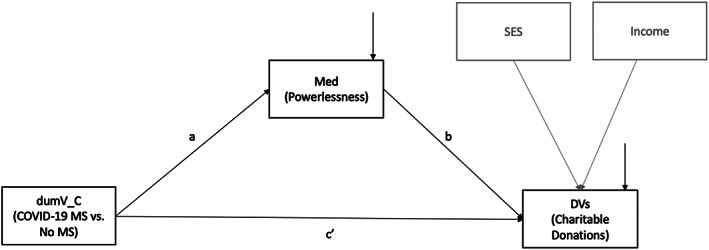
Study 2 (experiment): Hypothesized mediation model (H3). dumV_C is a dummy variable for condition (1 = COVID‐19 MS, 0 = no MS). All exogenous variables (dumV_C, SES, and Income) are allowed to covary with one another

The mediation hypotheses (H3) were tested in structural equation modeling (Figure 3) using the “lavaan” package (version 0.6–5). For the mediation effects, 95% bias‐corrected bootstrap confidence intervals were obtained from 1,000 bootstrap samples. All five mediation models fit the data well. The chi‐square statistics were 6.143 with *df* = 2 (*p* = 0.046), and RMSEA = 0.065 in all models. CFI ranged from 0.977 to 0.986. SRMR ranged from 0.029 to 0.030. The estimated path coefficients and mediation effects are reported in Table 3. The experimental condition had a significant (α = 0.05) mediation effect via powerlessness on monetary donation, volunteering time, and perceived value of time. Powerlessness was higher in the COVID‐19 MS condition than in No MS condition, and the higher Powerlessness led to higher responses in the dependent variables. To summarize, powerlessness mediated the effects of mortality salience manipulation on donation intentions (money and time) and monetary valuation of time, but not on the ratio of prosocial spending to proself spending. The proposed mediation model is presented in Figure 3 and the results of mediation analysis are shown in Table 3.

**TABLE 3:** Study 2 (experiment): Estimated path coefficients and mediation effects (H3)

Dependent variables	*c*'	*a*	*b*	Mediation effect (a*b)
Monetary donation ($)	6.336 (2.388)^**^	1.056 (0.135)^**^	2.896 (0.751)^**^	3.058 (1.481, 4.813)
Prosocial spending (ratio of spending on others to spending on self) (%)	3.360 (2.307)	1.056 (0.135)^**^	−0.120 (0.725)	−0.126 (−1.768, 1.274)
Volunteering time (hour)	0.620 (0.237)^**^	1.056 (0.135)^**^	0.425 (0.074)^**^	0.449 (0.271, 0.679)
Perceived value of time: Monetary valuation of time ($/hour)	1.914 (1.871)	1.056 (0.135)^**^	3.975 (0.588)^**^	4.197 (2.565, 6.270)
Charity involvement	0.634 (0.172)^**^	1.056 (0.135)^**^	0.066 (0.054)	0.070 (−0.035, 0.210)

*Note:* See Figure 3 for the hypothesized structural equation model. Powerlessness was the mediator in all models. For path coefficients c′, a, and b, unstandardized estimates are shown with *SE* in parentheses (**p < 0.01; *p < 0.05). For the mediation effects, unstandardized estimates are shown with 95% bootstrap confidence intervals in parentheses.

#### 5.3.3 Moderation effects of individual difference factors

**FIGURE 4:**
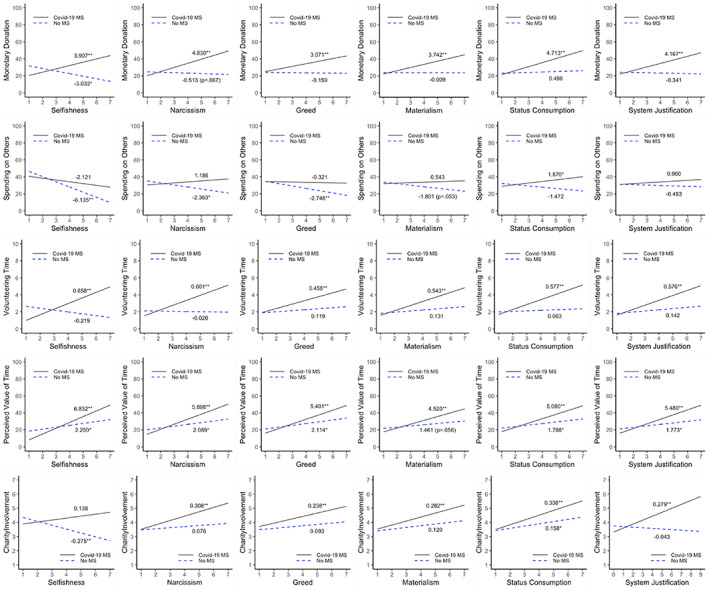
Study 2 (experiment): Interaction effects of experimental manipulation and dispositional factors (H5)

Regression analysis was conducted to test the interaction effects between the experimental condition and moderators (H5). Income and socioeconomic status (SES) were controlled since these factors need to be considered when investigating consumers' subjective well‐being (i.e., anticipated happiness and sense of power) in relation to macro‐economic health (Iyer & Muncy, 2016). Post hoc power analysis was conducted using an R package “pwr” (version 1.3‐0). With 487 observations, the regression analysis had 0.8 power to detect effect sizes as small as f^2^ = .0163 (i.e., *R* ^2^ = 0.0161). The estimated regression models are reported in Table 4. The interaction effects are depicted in the interaction plots and the estimated simple slopes for the moderator in each condition (COVID‐19 MS, No MS) are also shown in Figure 4. Supporting H5, the greedier, more selfish, narcissistic, materialistic, and system‐justifying the donor is, the higher the amounts of donations are, only when the COVID‐19‐induced mortality is made salient but not in the control condition.

**TABLE 4:** Study 2 (experiment): Estimated regression models for moderation effects (H5*)*

	Selfishness (H5a)	Narcissism (H5b)	Greed (H5c)	Materialism (H5d)	Status consumption (H5e)	System justification (H5f)
(intercept)	22.77 (1.61) **	23.48 (1.60)**	23.61 (1.67)**	23.68 (1.62)**	23.93 (1.64)**	23.51 (1.59)**
dumV_C	9.02 (2.35)**	8.01 (2.31)**	7.77 (2.41)**	7.52 (2.37)**	6.24 (2.37)**	7.81 (2.31)**
X	−3.03 (1.35)*	−0.51 (1.21)	−0.16 (1.08)	−0.01 (0.97)	0.50 (0.98)	−0.34 (0.96)
dumV_C * X	6.94 (1.90)**	5.34 (1.59)**	3.23 (1.43)*	3.75 (1.35)**	4.22 (1.30)**	4.51 (1.30)**
Interaction *R* ^2^	0.022	0.018	0.008	0.013	0.017	0.020
Model (DV: Monetary donation)	*R* ^*2*^ = 0.214, *F* = 26.14**	*R* ^*2*^ = 0.224, *F* = 27.77**	*R* ^*2*^ = 0.208, *F* = 25.29**	*R* ^*2*^ = 0.215, *F* = 26.34**	*R* ^*2*^ = 0.237, *F* = 29.94**	*R* ^*2*^ = 0.226, *F* = 28.09**
(intercept)	28.52 (1.52)**	29.62 (1.54)**	28.95 (1.59)**	29.54 (1.56)**	29.49 (1.59)**	30.20 (1.55)**
dumV_C	5.84 (2.21)**	3.62 (2.23)	4.88 (2.30)*	3.82 (2.28)	2.99 (2.30)	3.05 (2.25)
X	−6.14 (1.27)**	−2.36 (1.17)*	−2.75 (1.03)**	−1.80 (0.93)****	−1.47 (0.95)	−0.48 (0.94)
dumV_C * X	4.01 (1.78)*	3.55 (1.54)*	2.43 (1.37)****	2.34 (1.30)****	3.34 (1.26)**	1.44 (1.26)
Interaction *R* ^2^	0.009	0.010	0.006	0.006	0.013	0.003
Model (DV: Prosocial spending)	*R* ^*2*^ = 0.124, *F* = 13.62**	*R* ^*2*^ = 0.087, *F* = 9.15**	*R* ^*2*^ = 0.090, *F* = 9.51**	*R* ^*2*^ = 0.084, *F* = 8.85**	*R* ^*2*^ = 0.090, *F* = 9.54**	*R* ^*2*^ = 0.079, *F* = 8.27**
(intercept)	2.01 (0.16)**	2.06 (0.16)**	2.14 (0.17)**	2.13 (0.16)**	2.10 (0.16)**	2.12 (0.16)**
dumV_C	0.92 (0.23)**	0.88 (0.23)**	0.75 (0.24)**	0.74 (0.23)**	0.68 (0.24)**	0.78 (0.23)**
X	−0.22 (0.13)	−0.03 (0.12)	0.12 (0.11)	0.13 (0.10)	0.06 (0.10)	0.14 (0.10)
dumV_C * X	0.88 (0.19)**	0.63 (0.16)**	0.34 (0.14)*	0.41 (0.13)**	0.51 (0.13)**	0.43 (0.13)**
Interaction *R* ^2^	0.033	0.024	0.009	0.015	0.024	0.017
Model (DV: Volunteering time)	*R* ^*2*^ = 0.254, *F* = 32.81**	*R* ^*2*^ = 0.260, *F* = 33.82**	*R* ^*2*^ = 0.249 *F* = 31.96**	*R* ^*2*^ = 0.261, *F* = 34.09**	*R* ^*2*^ = 0.278, *F* = 37.09**	*R* ^*2*^ = 0.277, *F* = 36.89**
(intercept)	25.14 (1.26)**	25.04 (1.25)**	25.50 (1.28)**	25.11 (1.28)**	25.43 (1.29)**	25.08 (1.23)**
dumV_C	3.12 (1.83)	3.55 (1.81)****	2.01 (1.85)	3.15 (1.87)	1.96 (1.86)	3.15 (1.79)****
X	2.25 (1.05)*	2.09 (0.95)*	2.11 (0.83)*	1.46 (0.76)****	1.79 (0.77)*	1.77 (0.75)*
dumV_C * X	4.58 (1.48)**	3.81 (1.25)**	3.29 (1.10)**	3.06 (1.07)**	3.29 (1.02)**	3.71 (1.00)**
Interaction *R* ^2^	.015	.015	.014	.014	.017	.021
Model (DV: Monetary valuation of time)	*R* ^*2*^ = 0.227, *F* = 28.22**	*R* ^*2*^ = 0.233, *F* = 29.33**	*R* ^*2*^ = 0.243, *F* = 30.87**	*R* ^*2*^ = 0.211, *F* = 25.69**	*R* ^*2*^ = 0.244, *F* = 31.00**	*R* ^*2*^ = 0.253, *F* = 32.58**
(intercept)	3.55 (0.12)**	3.65 (0.11)**	3.68 (0.12)**	3.69 (0.12)**	3.72 (0.12)**	3.61 (0.11)**
dumV_C	0.74 (0.17)**	0.58 (0.17)**	0.53 (0.17)**	0.51 (0.17)**	0.41 (0.17)*	0.61 (0.17)**
X	−0.28 (0.10)**	0.08 (0.09)	0.09 (0.08)	0.12 (0.07)	0.16 (0.07)*	−0.04 (0.07)
dumV_C * X	0.41 (0.14)**	0.23 (0.11)*	0.15 (0.10)	0.16 (0.10)	0.18 (0.09)****	0.32 (0.09)**
Interaction *R* ^2^	0.016	0.007	0.004	0.005	.006	0.021
Model (DV: Charity involvement)	*R* ^*2*^ = 0.166, *F* = 19.09**	*R* ^*2*^ = 0.176, *F* = 20.51**	*R* ^*2*^ = 0.171, *F* = 19.81**	*R* ^*2*^ = 0.180, *F* = 22.06**	*R* ^*2*^ = 0.204, *F* = 24.66**	*R* ^*2*^ = 0.181, *F* = 21.21**

*Note:* Unstandardized coefficients are shown with *SE* in parentheses. Moderator (X) is centered at the sample mean. dumV_C is a dummy variable for condition (1 = COVID = 19 MS, 0 = No MS). SES and Income were controlled for (coefficient estimates are not shown in the table). Interaction *R* ^2^ is the R square increment attributable to the interaction effect. The degrees of freedom for F statistic was (5, 481).

^****^ *p* < 0.08; ^***^ *p* < 0.001; ^**^ *p* < 0.01; ^*^ *p* < 0.05.

## 6 GENERAL DISCUSSION

### 6.1 Key findings and theoretical contributions

Despite the burgeoning literature informing implications of terror management theory for a wide range of human cognition, emotion, and behavior, little attention has been paid to how people respond to epidemics and pandemics. The current research, which entails a sense of immediacy and urgency, addresses the timeliest issue of the COVID‐19 pandemic drawing from an evolutionary terror management perspective. It marks the first test of (a) the integrative model consisting of death‐thought accessibility hypothesis, mortality salience hypothesis, and anxiety buffer hypothesis, in light of the COVID‐19 pandemic; and (b) the interaction effects of mortality salience and donors' individual difference factors on the paradox of egocentrism and altruism, during the currently occurring pandemic.

This research proposes that charitable giving and prosocial (as opposed to proself) spending during the pandemic are evolutionarily adaptive coping strategies to buffer against death anxiety and that the COVID‐19‐induced mortality salience increases people's need for this buffer. Using both a survey design (Study 1) and an experimental design (Study 2), this multi‐method study addresses the dual defense model of mortality salience, an extension of terror management theory (Arndt et al., 2005; Arndt, Solomon, Kasser, Kennon, & Sheldon, 2004; Pyszczynski et al., 2000), which distinguishes between mortality salience effects driven by unconscious accessibility of death‐related thoughts (i.e., distal defenses) and those driven by conscious accessibility of death‐related thoughts (i.e., proximal defenses) (Maheswara & Agrawal, 2004; Pyszczynski et al., 1999). The non‐significant and negative path from death‐thought accessibility to powerlessness and materialism in Study 1 (survey data) implicitly supports the notion of unconscious accessibility and distal defenses, while the significant main effect of the explicit COVID‐19‐induced mortality salience manipulation on death‐thought accessibility in Study 2 (experimental data) explicitly supports the notion of conscious accessibility and proximal defenses. The key finding across the two studies is: people intend to donate more and spend more on others when people feel fearful, whether they are anxious about death (unconscious, distal defense in Study 1) or mortality is experimentally activated and made salient (conscious, proximal defense Study 2). Study 2 is the first empirical evidence about the causal relationship between the experimentally activated COVID‐19‐induced mortality salience on charitable giving and prosocial spending, as anxiety buffering coping strategies.

The current research makes several theoretical and methodological contributions to the terror management and system justification literature by addressing the research trajectories suggested in the provocative dialogues among scholars committed to research on terror management account of materialism and consumer behavior (Arndt, Solomon, Kasser, Kennon, & Sheldon, 2004; Maheswara & Agrawal, 2004; Rindfleisch & Burroughs, 2004) as well as system justification explanations of mortality salience and powerlessness (Jost, 2019; Landau et al., 2004; Van Der Toorn et al., 2015). First, it is worth noting that mortality salience experimentally activated via exposure to the COVID‐19‐related media content amid the pandemic contributes to people's social system justification within a capitalist, materialistic, market economy, despite the seemingly unfair impact of social class, poverty, and economic inequality on the severity level of the pandemic among the underprivileged population. This finding is not only compatible with the social system justification theory, which proposes that powerlessness fosters legitimation of inequality and increases system justification even when inequality in the system was made explicitly salient (Jost, 2019; Jost et al., 2003) but also further confirms the anxiety buffering and worldview defense hypothesis of terror management theory, which posits that people reinforce their cultural worldview in face of mortality salience (Greenberg et al., 1997). Departing from the previous mortality salience subtle manipulation paradigm, the current experiment manipulated mortality salience explicitly by exposing the participants in the treatment condition to ecologically valid media content about the COVID‐19‐induced mortality rate and death toll currently relevant to our society.

Second, the existing literature on charitable donations (Cha et al., 2020) and on the association between mortality salience and charitable behaviors (Jonas et al., 2013) heavily focused on monetary donation, while relatively scant attention has been paid to time donation. The present study addresses this gap by examining multiple dimensions of donation intentions including monetary donation (absolute amount of $), the ratio of prosocial to proself spending (relative %), donation of time (hours spent on volunteering), monetary valuation of donors' own time (hourly rate), economic value of the donated time (hourly rate x hour), and the relative valuation of time versus money. Consistent results across these multiple dimensions speak to the robustness of the current findings. This research advances theory by adding a new mediation mechanism (powerlessness) and individual difference factors (selfishness, narcissism, greed, materialism, and system justification tendency) as boundary conditions for the proposed relationship between mortality salience and charitable giving, to the terror management literature and the fundraising literature on time‐versus‐money valuation.

Last, and most importantly, the present research examined the moderating roles of individual difference and personality factors in the connection between mortality salience and charitable donations, as one of important monetary decision‐making behaviors (Jonas et al., 2013), in the relevant context of the COVID‐19 pandemic. To this end, the experiment tested the original proposition that the extent to which mortality salience increases charitable giving and prosocial spending depends upon people's selfishness (Study 2 H5a), narcissism (H5b), greed (H5c), materialism (H5d), status consumption tendency (H5e), and system justification tendency (H5f) as boundary conditions. One aspect that has received less attention within the terror management literature is the interaction effects of mortality salience and individual difference factors such as personalities and value systems. The present study addresses this gap by stimulating theoretical discussions about the moderating effects of relevant personality/dispositional trait factors (selfishness, narcissism, and greed) and value systems (materialism, need for status manifested by conspicuous status consumption, and system justification) and providing rich empirical data supporting the original theoretical propositions. Ultimately, these moderation effects explain the paradox of egocentrism and altruism under death threat by showing that greedier, more selfish, narcissistic, materialistic, and system‐justifying people donate greater amounts of time and money when mortality salience is made salient.

### 6.2 Practical implications

This research may provide several practical implications for social marketers, policy makers, and consumer advocacy groups as well as for health communication, crisis management, and the science of fundraising.

Research on consumer affairs should investigate how consumption affects consumer well‐being in order to empower them to live happier lives (Iyer & Muncy, 2016; Nepomuceno & Laroche, 2016). In Study 2, consumers' income and SES (socio‐economic status) were controlled to consider not only subjective well‐being (i.e., anticipated happiness and sense of power) but also more traditional economic measures to assess macro‐economic health (Diener et al., 2008; Iyer & Muncy, 2016). This academic study sought to examine empirically the potential effectiveness of mortality salience priming in changing consumers' attitudes, intention to donate (time and money), and intention to spend money on others, advocated by social marketers. As noted by Kareklas and Muehling (2014), “the use of death symbols in public service announcements (PSAs) is easy and inexpensive to implement, thereby increasing their applicability by social marketers” (p. 244). In line with this paradigm, the current findings can provide insights and practical guidelines for social marketers and policy makers endeavoring to encourage consumers' prosocial behaviors like spending on others and giving back to the society by empirically demonstrating that mortality salience primes could be an effective strategy for social marketers and policy makers to consider.

Previously, anti‐materialism and consequent anti‐consumption behaviors were perceived as “a challenge for marketers and managers due to their emphasis on rejecting consumption” (Lee & Ahn, 2016, p. 42). Also, anti‐consumption lifestyles are considered incompatible with economic welfare and consumer well‐being in a consumerist society (Balderjahn et al., 2020). However, donation behaviors and prosocial *spending* can be considered as “experiential spending” (as opposed to “proself spending for material possession”). Approaching prosocial spending and donation behaviors from the perspective of experiential spending can have important implications for marketing managers and social marketers in terms of targeting not only voluntary simplifiers with anti‐consumption values who reject consumption (Lee & Ahn, 2016; Nepomuceno & Laroche, 2016) but also greedy and material consumers, thus leveraging current study's empirical data to promote greater donation behaviors. Furthermore, social marketers and policy makers can highlight not only psychological benefits such as happiness and sense of power, which were investigated in the current study, but also practical benefits from giving when targeting greedy and material consumers, such as “tax breaks and financial credits available to donors” (Sarofim et al., 2020, p. 1032) as strategic methods of creating appeals and incentives, thus ultimately promoting greater donation behaviors. On the one hand, the current findings may better equip social marketers to deliver intangible value to *donors* in the form of happiness and sense of power (psychological/subjective well‐being) by fostering donation behaviors especially during circumstances in which death‐threats are primed and the notion of mortality is made salient. On the other hand, the current study implies that social marketers, policy makers, and consumer advocacy groups can also exert a positive influence on the *recipients* (economic/financial well‐being via monetary donation and psychological/subjective well‐being via time donation) by evoking death‐thoughts and priming mortality salience, thus prompting people to *donate* time and money. Consumer advocates and policy makers need to have a solid understanding of *what* influences *which* type of consumption behavior (e.g., prosocial versus maladaptive consumer behavior) (Boland et al., 2020).

With regard to health communication (Rubinelli et al., 2020) and health marketing (Karasneh et al., 2021), this research suggests that death anxiety amplified and mortality made salient during the pandemic can be catalysts that prompt people's behavioral intentions to engage in health‐related prosocial behaviors. Mortality salience priming for health risk messages in public service advertisements and campaigns in the realm of public health is an inexpensive and effective strategy to persuade consumers, especially given the challenges faced by public policy makers to increase the effectiveness of health communication campaigns. For example, even accidental exposure to media contents and/or PSAs covering a wide range of topics and news stories that bring mortality salience closer to consciousness such as the COVID‐19 death toll, mortality rate amplified for underserved communities with lower socioeconomic status, and physical distancing/self‐quarantine practice to lower infection rate and mortality rate, and so forth may increase (a) not only people's monetary donations to CDC (Center for Disease Control and Prevention, 2020) Foundation and the amount of time they, as “disaster relief volunteers,” are willing to spend on volunteering for CDC (b) but also the general public's intention to comply with COVID‐19 preventive measures, rules, and regulations. In addition, the importance of risk management and effective crisis communication cannot be overstated during the COVID‐19 pandemic (Abrams & Greenhawt, 2020). Findings from the current study implicates that exposure to the COVID‐19‐related media content and/or PSAs can have differential effects on people's behavioral intention during the risk management and crisis communication process.

With regard to implications for the art and science of fundraising and donation appeals, the current findings speak to not only the efficacy of fear appeals in increasing charitable donations but also the importance of charitable donor market segmentation with regard to the level of donations and methods of prompted giving. Identifying different characteristics of donors and accurate targeting of likely donors are vital for streamlining fundraising strategies and charities' survival (Schlegelmilch et al., 1997). Although prior research confirmed the positive impact of mortality salience on the efficacy of donation appeals (Cai & Wyer, 2015), the current research provides even more specific suggestions as to how to develop and utilize charity market segmentation strategies for fundraising and donation appeals: charities may highlight the COVID‐19‐related mortality salience such as death toll statistics when targeting donors who are selfish, narcissistic, greedy, and materialistic, as well as who have higher need for status, status consumption tendency, and system‐justifying tendency. Based on the analysis of donor profiles (e.g., income, socioeconomic status, hourly rate, etc.) and donation history regarding donation method preference and types/amounts of charitable giving (e.g., anonymized donation versus publicized donation, time versus monetary donation, one‐time versus monthly donation, etc.), it is feasible to segment the donor pool and customize donation appeals for solicitation. There are also many other factors that motivate charitable giving such as donation facilitation process and donor benefits (Whillans, 2016). For example, given that the happiness benefits of helping others are magnified when the charitable appeal highlights the tangible impact of their donation (Aknin et al., 2013), specifying the real impact of donation on the life of recipients would help amplify the impact of including mortality salience and fear appeals on charitable donations during the COVID‐19 pandemic.

### 6.3 Study limitations as a springboard for future research

Despite noteworthy strengths including measurement of multiple aspects of charitable donations (time, money, monetary valuation of time, economic value of donated time, ratio of prosocial to proself spending, etc.), moving beyond undergraduate samples, theoretical advancement of the terror management literature by adding a new mediating mechanism (powerlessness) and moderators (dispositional factors) as boundary conditions, to name a few, the current research is not without limitations. Several limitations of the current study can be discussed with regard to constructive suggestions for this line of future research.

First, there are several caveats that need to be discussed with regard to generalizability of the current findings. Donation intentions may differ from actual donation behavior (Baumeister et al., 2007; Cha et al., 2020). Due to the stay‐home‐orders, school closures, and social distancing/physical distancing, data collections were conducted via an online survey and an online experiment, which limits ecological isomorphism of donation behaviors in the offline setting. Once the shelter‐in‐place order is lifted, (a) an offline field experiment with real donation appeals will enhance realism and external validity and (b) face‐to‐face in‐depth interviews will provide deeper insights into people's real motivation for prosocial behaviors under death threat. Furthemore, the cross‐sectional nature of survey data and one‐time experimental snapshots of the outcomes of mortality salience limit the power of this research in explaining *change*s in consumers' cognition, emotion, and behavior over the long‐term course of the pandemic. Follow‐up research needs to be supplemented by longitudinal studies in order to uncover the complex coping process as suggested by Rindfleisch and Burroughs (2004) as well as to examine how consumers dynamically navigate and respond to death threat at different developmental stages and lifting phases of the pandemic (Mendy et al., 2020). In addition, the “novelty” factor of this particular virus (COVID‐19 also known as, literally, *Novel* Coronavirus) might have played either a moderating or a mediating role in forming consumers' perceived fear, sense of power (versus powerlessness), and the amount of death‐thoughts generated, etc. compared to other types of even more deadly viruses such as Ebola virus, SARS, MERS, and HIV/AIDS without the novelty element any more. Replications of the current cross‐sectional survey and between‐subjects experiment examining consumers' reaction to and perception of these different types of viruses and their impact on donation behaviors will be needed to accurately evaluate the degree to which the current findings can be generalizable. Relatedly, the current study specifically focuses on the application of terror management theory to “pathogen” threats in order to address relevant topics revolving around the special issue's central theme of global‐level *pandemics* and consumer well‐being. Follow‐up studies need to further compare between different *types* of threats (e.g., gun threat versus pathogen threat) and their impact on donation intention and consumer well‐being. Also, there are not only a wide variety of *types* of death threats but also at different *units* or *levels* of analysis such as cancer diagnosis at the individual/family level (Fisher & Nussbaum, 2015), crisis management during the pandemic at the organizational level (Wong et al., 2021), natural disasters at the national level (Baker, 2009), man‐made calamities like military clash at the international level (Kimhi et al., 2020), terrorist attacks at the international/intercultural level (Das et al., 2009; Fischer‐Preßler et al., 2019), among others. Follow‐up studies about terror management need to examine different *types* of death threats at different *units/levels* of analysis and their impact on consumer well‐being.

Second, data were collected among Americans who reside in the US, in order to test the anxiety buffering hypothesis within a capitalistic society. Consequently, the roles of different cultures (e.g., individualistic versus collectivistic) and economic systems (e.g., capitalist free market economy versus socialist market economy) were not directly tested. Replication of the current survey and experiment in other countries may yield different results and provide deeper insights into the role played by cultural standards, norms (Jonas et al., 2013), economic systems, and worldviews. Furthermore, this research only examined the effects of mortality salience on instant and short‐term donation intentions. Donations with long‐term sustainability and lasting impact (e.g., “donate once” versus “donate monthly”) need to be examined in follow‐up studies with longitudinal design. With regard to donation intentions, the current study did not measure consumers' “money attitude” (Pereira & Coelho, 2019). More specifically, the *power* dimension of the participants' money attitude, which represents “a concern for money as an instrument to gain power over others and as a symbol of status and success” (Pereira & Coelho, 2019, p. 427) might have played a significant mediating role in determining the effects of mortality salience on donation intentions. Additionally, the current study is missing discussion about *recipients'* response to charitable giving (Zhang et al., 2018; Zhang et al., 2020), while focusing only on prosocial spending from the *givers'* perspective. While a growing body of studies has explored the positive relationship between prosocial spending and spenders' happiness, happiness of recipients has been frequently ignored (Zhang et al., 2018; Zhang et al., 2020). This gap needs to be further addressed in follow‐up studies by applying the terror management theory such that how *recipients* respond to and manage terror/crisis and how proposical spending influences the *recipients'* well‐being. With regard to the *recipients* (specifically “to where/to whom” participants' time and money would be donated in light of the pandemic), we measured consumers' donation intention in general using a generic language without narrowing down the recipient of participants' donation. In other words, we did not specify the recipient for the sake of ensuring a decent level of generalizability of our findings about consumers' donation intention and their sense of psychological well‐being fostered by donation in response to COVID‐19‐induced death threat. We attempted to ensure a decent level of generalizability of our findings with regard to donation intention, since the novelty element of the COVID‐19 and exclusion of other types of death threat or uncertainty such as cancer diagnosis from our manipulation stimuli already limit generalizability of the current findings, as discussed above. Nonetheless, follow‐up studies need to measure consumers' intention to donate to specific individuals/organizations relevant to the types/levels of death threats (e.g., cancer threats‐American Cancer Society paired up, natural disaster‐relief organizations paired up, etc.). Furthermore, the current research heavily focuses on consumers' donation‐related psychology and behavior at the individual level, while disregarding corporate strategic giving (Aitken & Watkins, 2017) at the organizational level. It would be also important to study the impact of corporate strategic giving not only on consumers' well‐being but also on consumers' reaction to corporate social responsibility (CSR) amid the COVID‐19 pandemic.

Third, the role of religion was not considered despite the notion that death anxiety motivates people to employ a broad range of cultural coping mechanisms such as materialism, religion, and patriotism (Becker, 1973). Beliefs about and orientation toward deaths vary with religious motivation and religious belief salience (Christopher et al., 2006). The variations between and among different religions (e.g., Christianity, Buddhism, Islam, Hinduism, and Jusaism, etc.) with regard to orientation toward death, system justification, and materialistic possessions might have played a role in determining the effects of mortality salience on prosociality and charitable giving. Follow‐up studies certainly need to measure participants' religion to provide deeper insights into the role of religion in coping with existential insecurity induced by life‐threatening pandemics. In addition, this study heavily focused on biological death threat while not considering financial threat resulting from economic crisis and job loss which might correlate with materialism and system justification (McCabe & Daly, 2018). Follow‐up studies need to examine the influence of religion on the *financial well‐being* (defined as “a state of being financially healthy, happy, and free of worry”) of consumers (Sarofim et al., 2020, p. 1031), drawing from terror management theory. For example, graduating during an economic recession has been correlated with higher levels of materialism (Sheldon & Kasser, 2008). As “the health crisis metastasizes into an economic crisis” (Mendy et al., 2020), follow‐up studies need to measure employment insecurity and fear of job loss as covariates.

Fourth, this study did not explicitly measure people's loneliness and social isolation resulting from the physical distancing, which might have played a role in accounting for people's donation intentions, especially the amount of time they are willing to spend on helping others and volunteering for a charity, which involve some level of social connection. Relatedly, research shows that valuing time over money is associated with needs for social connection (Willans & Dunn, 2019). Also, investigation of the relationship between the *recipients*' facilitated sense of social connection and advanced happiness (Bastos, 2020) resulting from the *givers*' donation will be a meaningful addition to this line of research on the recipients' both financial well‐being and psychological well‐being fostered by both *monetary donation* (money) and *experiential donation* (time). Building upon the current data that confirms the higher perceived value of donated time (i.e., economic value of donated time) compared with the amount of donated money when mortality salience is made salient, this line of future research needs to examine the extent to which people value their time they spend with others, in relation to the social/physical distancing experience before, during, and after the pandemic. Pre‐peri‐post‐pandemic comparisons regarding public perception of social distancing and its impact on monetary valuation and economic value of socializing deserve additional empirical testing.

### 6.4 Epilogue

The present study endeavors to provide insights into terror management and system justification during the COVID‐19 pandemic by examining why and how the current crisis makes vulnerability to death salient and poses a threat to people's sense of meaning, which prompt people's need for biological survival as an animal and need for meaning‐making as a social and cultural animal. This research explored how environmental situational factors and dispositional personality factors jointly influence vulnerability to mortality salience and consequent cognitive, affective, and behavioral outcomes.

People reveal their true colors in face of adversity and crisis. In light of the current study's empirical findings that sadly reinforce the social system justification within a capitalist, materialistic, market economy in spite of the seemingly unfair impact of the COVID‐19 pandemic on social class, poverty, and economic inequality, the poor and vulnerable populations “at the bottom of the global economic pyramid” (Hill & Sharma, 2020; Martin & Hill, 2012) must not be forgotten by governments, policy makers, consumer advocacy groups (e.g., Center for Science in the Public Interest, Consumer Union, Alliance for Justice, etc.), and businesses in this time of need (Hill, 2020). Nonetheless, the findings from this research are still encouraging and give us hope that we can revitalize resilience (Huang et al., 2020), referring to “a pattern of positive responses to an adverse situation or crisis that is sufficient for optimizing human potential” (Herdman, 2015, p. 376) and contribute to the robustness of humankind's capabilities to cope with emergencies. Amid the COVID‐19 pandemic, we make a life by what we give: not only others' lives but also our own lives.
